# Hydrogen and carbon isotope fractionation during degradation of chloromethane by methylotrophic bacteria

**DOI:** 10.1002/mbo3.124

**Published:** 2013-09-08

**Authors:** Thierry Nadalig, Markus Greule, Françoise Bringel, Stéphane Vuilleumier, Frank Keppler

**Affiliations:** 1 Equipe Adaptations et Interactions Microbiennes dans l'Environnement, UMR 7156 Université de Strasbourg - CNRS28 rue Goethe, Strasbourg, 67083, France; 2 Max Planck Institute for ChemistryHahn-Meitner-Weg 1, 55128 Mainz, Germany

**Keywords:** Carbon isotope fractionation, chloromethane biodegradation, hydrogen isotope fractionation, methylotrophic bacteria

## Abstract

Chloromethane (CH_3_Cl) is a widely studied volatile halocarbon involved in the destruction of ozone in the stratosphere. Nevertheless, its global budget still remains debated. Stable isotope analysis is a powerful tool to constrain fluxes of chloromethane between various environmental compartments which involve a multiplicity of sources and sinks, and both biotic and abiotic processes. In this study, we measured hydrogen and carbon isotope fractionation of the remaining untransformed chloromethane following its degradation by methylotrophic bacterial strains *Methylobacterium extorquens* CM4 and *Hyphomicrobium* sp. MC1, which belong to different genera but both use the *cmu* pathway, the only pathway for bacterial degradation of chloromethane characterized so far. Hydrogen isotope fractionation for degradation of chloromethane was determined for the first time, and yielded enrichment factors (ε) of −29‰ and −27‰ for strains CM4 and MC1, respectively. In agreement with previous studies, enrichment in ^13^C of untransformed CH_3_Cl was also observed, and similar isotope enrichment factors (ε) of −41‰ and −38‰ were obtained for degradation of chloromethane by strains CM4 and MC1, respectively. These combined hydrogen and carbon isotopic data for bacterial degradation of chloromethane will contribute to refine models of the global atmospheric budget of chloromethane.

## Introduction

Chloromethane (CH_3_Cl) is the most abundant volatile halocarbon in the atmosphere and is responsible for about 16% of chlorine-catalyzed ozone destruction in the stratosphere (Montzka et al. 2011). Major sources of CH_3_Cl are natural, and include tropical forests, grasslands, salt marshes, peatlands, biomass burning, and oceans. Most atmospheric CH_3_Cl is released from terrestrial vegetation (Yoshida et al. 2004; Keppler et al. 2005), with a fraction suggested to be formed in dead and senescent leaf tissue (Hamilton et al. 2003). Higher plants, especially the phyllosphere (i.e., the aboveground part of vegetation), represent the major biotic source of atmospheric chloromethane (Saito and Yokouchi 2008).

The global sink of chloromethane is estimated at 4500–5500 Gg year^−1^ (Khalil and Rasmussen 1999), the predominant loss process being by reaction with hydroxyl radicals in the troposphere (Yoshida et al. 2004). However, degradation of CH_3_Cl may also occur biologically through chloromethane-degrading bacteria, which can use this compound as their sole source of carbon and energy for growth. Such bacteria were isolated from soils, polluted industrial sites, activated sludge, freshwater, oceans, and most recently from the phyllosphere (Hartmans et al. 1986; Doronina et al. 1996; Coulter et al. 1999; McAnulla et al. 2001; Schäfer et al. 2005; Nadalig et al. 2011). The only known pathway for aerobic chloromethane degradation, termed *cmu* for chloromethane utilization, is specific for halogenated methanes (CH_3_Cl, CH_3_Br, CH_3_I) and was fully characterized for strain *Methylobacterium extorquens* CM4. Dehalogenation of chloromethane involves two genes encoding methyltransferases associated with corrinoid and folate cofactors (Vannelli et al. 1998, 1999; Studer et al. 1999, 2001, 2002; Roselli et al. 2013) (see Fig. S1). Fully assembled genome sequences are available for chloromethane-degrading strains *M. extorquens* CM4 (Marx et al. 2012) and *Hyphomicrobium* sp. MC1 (Vuilleumier et al. 2011), facilitating the detailed analysis of chloromethane metabolism at the molecular level.

Although it was recently established that terrestrial ecosystems play an important role in production and consumption of chloromethane, corresponding sources and sinks remain poorly characterized (Rhew 2011). Stable isotope analysis, used in combination with chloromethane flux measurements, has the potential to constrain the atmospheric chloromethane budget (Gola et al. 2005; Keppler et al. 2005) and can be applied to help resolve uncertainties regarding chloromethane sources and sinks. Due to the isotope effect, which bases on the fact that chemical bonds between heavier isotopes break more slowly than bonds between lighter ones (primary isotope effect), isotope fractionation occurs whenever chloromethane is degraded. Furthermore, the isotopic nature of atoms adjacent to the broken bond may also contribute to chemical reactivity (secondary isotope effect; Elsner et al. 2005). Different isotope ratios result from both isotope effects and may thus be helpful to distinguish different sources and sinks for a given compound of interest (see Elsner et al. (2005) for a more detailed treatment of stable isotope fractionation). Stable carbon isotope signatures (ratio of ^13^C/^12^C or δ^13^C values) of chloromethane sources were determined for forest soils (Redeker and Kalin 2012), plants (Harper et al. 2003; Keppler et al. 2004), fungi (Harper et al. 2001), and for emissions resulting from biomass burning (Rudolph et al. 1997; Czapiewski et al. 2002). Stable carbon isotope modeling studies (Keppler et al. 2005; Saito and Yokouchi 2008) support the contention that a substantial bacterial sink (>1000 Gg year^−1^) is involved in the global chloromethane budget (Miller et al. 2001, 2004; Harper and Hamilton 2003). The only study of isotope fractionation resulting from bacterial chloromethane degradation so far was performed with methylotrophic bacterial strains *Aminobacter ciceronei* IMB-1 and *Aminobacter lissarensis* CC495 (Miller et al. 2001). The authors found closely similar values for both strains for the enrichment in ^13^C of the remaining untransformed chloromethane.

In this study, we determined the hydrogen isotopic fractionation of remaining untransformed chloromethane following bacterial degradation, and compared it with measurements of carbon isotopic fractionation for two well-characterized chloromethane-degrading strains, *M. extorquens* CM4 (Doronina et al. 1996; Marx et al. 2012; Roselli et al. 2013) and *Hyphomicrobium* sp. MC1 (Hartmans et al. 1986; Vuilleumier et al. 2011).

## Material and Methods

### Microorganisms and cultivation

Strains *M. extorquens* CM4, *M. extorquens* AM1, and *Hyphomicrobium* sp. MC1 were laboratory stocks and cultivated in a mineral medium for methylotrophic bacteria (M3) containing (L^−1^ of distilled water) KH_2_PO_4_ (6.8 g), (NH4)_2_SO_4_ (0.2 g), NaOH (5 mol L^−1^ ) (5.85 mL), yielding a final pH of 7.2. After autoclaving, 1 mL L^−1^ medium each of calcium nitrate solution (25 g L^−1^), trace elements solution containing (mg L^−1^) FeSO_4_^.^7H_2_O (100), MnSO_4_^.^H_2_O (100), ZnSO_4_ (29.5), Co(NO_3_)_2_^.^6H_2_O (25), CuCl_2_^.^H_2_O (25), Na_2_MoO_4_^.^2H_2_O (25), NH_4_VO_3_ (14.4), NiSO_4_^.^6H_2_O (10), H_3_BO_3_ (10), and 0.5 mL-L^−1^of H_2_SO_4_ (95%) were added. Strains CM4 and MC1 were grown with chloromethane gas (10 mL gas, effectively giving approximately 10 mmol L^−1^, Fluka, Buchs, Switzerland) in 300-mL Erlenmeyer vials fitted with sealed mininert valve caps (Supelco, Sigma-Aldrich France, Lyon, France) and containing 50 mL of M3 medium. Strain AM1 was grown with methanol (10 mmol L^−1^) as sole carbon source. All cultures were incubated at 30°C on a rotary shaker (100 rpm).

### Cell suspensions

Cultures were harvested in late exponential phase of growth (OD_600 nm_ ∼0.5) and washed twice with 50-mL phosphate buffer pH 7.0. Cell pellets were resuspended in 25 mL of the same buffer in 300-mL Erlenmeyer vials fitted with a sealed mininert valve cap (Sigma). Chloromethane gas (5 mL, 10 mmol L^−1^) was added with a syringe through the septum, and vials were incubated at 30°C with shaking (100 rpm). Abiotic controls (no cells added) and controls with strain *M. extorquens* AM1 unable to degrade chloromethane were prepared and incubated in the same way. The headspace was sampled every hour (0.1 mL) for determination of chloromethane concentration by gas chromatography, and 1-mL headspace samples were also taken every 30 min and conserved in 12 mL Exetainers® (Labco Limited, Lampeter, UK) for subsequent isotopic measurements. At the end of incubation experiments, duplicate 1-mL samples of cell suspensions were transferred to Eppendorf tubes and used for determination of protein concentration using the bicinchonic assay (Sigma) with bovine serum albumin used as a standard. Concentration of chloride was measured in supernatants of cell suspensions using the spectrophotometric method of Jörg and Bertau (2004).

### Gas chromatography

Chloromethane was quantified using a CP 3800 gas chromatograph connected to a flame ionization detector (GC-FID; Agilent Technologies France SAS, Courtabeuf, France). Headspace samples (100 μL) of cell suspensions were collected with a gastight 1750 syringe (Hamilton Bonaduz AG, Bonaduz, Switzerland) and injected onto the GC column (CP-Sil 5 CB, length 15 m; Varian). Separation of volatile compounds was achieved by isothermal elution at 30°C for 1 min, followed by a linear gradient of temperature increase to 220°C at 20°C min^−1^. Injector and detector were maintained at 220°C (splitless mode) and 300°C, respectively; makeup gas was nitrogen (N_2_; Linde Gas). Peak areas were analyzed with Galaxie Workstation software (Varian).

### Gas chromatography isotope ratio mass spectrometry

#### Cryogenic preconcentration unit

A cryogenic preconcentration unit with several purification and concentration steps was designed and built in-house. For a detailed description of the system as well as a graphical presentation refer to Greule et al. (2012). Briefly, following evacuation of a sample loop (100 mL), a gas sample is allowed to enter the sample loop after passing through an Ascarite II chemical trap for removal of CO_2_. Following equilibration, the sample is flushed into two physical traps. The temperature of the first trap (empty 1/8″ stainless steel tube) is set at −30°C to remove water. The second trap (1/8″ stainless steel tube filled with glass beads) is cooled to −170°C, a temperature sufficient to trap CH_3_Cl quantitatively. After 10 min, this trap is rapidly heated to 150°C and CH_3_Cl is flushed to a focus trap (50 cm × 0.25 mm i.d. deactivated fused silica capillary placed in liquid nitrogen, −196°C). After 15 min, the focus trap is raised out of liquid nitrogen permitting the transfer of CH_3_Cl to the gas chromatography–stable isotope ratio mass spectrometry (GC-IRMS) unit via a heated transfer line (90°C).

To retain comparability between measurements, all samples were analyzed using the same system, even if no preconcentration was necessary. Depending on the sample concentration of chloromethane, an aliquot from the Exetainers® (see section “Cell suspension” above) was transferred to a 100-mL-sealed glass vessel (before hand flushed with nitrogen) to reach a mixing ratio of about 10ppm CH_3_Cl. The glass vessel was subsequently connected to the preconcentration unit. Exetainers® containing samples with lower CH_3_Cl concentration were directly connected to the preconcentration unit.

#### GC-IRMS analysis of δ^2^H of chloromethane

Hydrogen isotope ratios of CH_3_Cl (δ^2^H_CH3Cl_) were measured by compound specific high-temperature conversion isotope ratio mass spectrometry analysis (GC-TC-IRMS). The cryogenic preconcentration unit described above was directly coupled to a Hewlett Packard HP6890 gas chromatograph (Agilent Technologies, Palo Alto, CA), interfaced with an Isoprime IRMS via a 1050°C GCV high-temperature conversion reactor (Isoprime, Manchester, UK). The GC was equipped with a GasPro column (60 m × 0.32 mm i.d.; Agilent Technologies) and was held isothermal at 150°C. The pyrolysis reactor contained a 0.65 mm i.d. quartz tube packed with chromium pellets (Isoprime). The helium flow coming from the preconcentration unit was set to 1.0 mL min^−1^.

A tank of ultrahigh-purity hydrogen (Hydrogen 5.0; Air Liquide, Düsseldorf, Germany) with a certified δ^2^H value of −172‰ ± 2‰ (certified by Air Liquide) was used as the working reference gas. The H_3_^+^ factor, determined daily during this investigation (3-month period), was in the range 3.96–4.37.

Throughout this study, the conventional “delta” notation, which expresses the isotopic composition of a material relative to that of a standard on a per mil (‰) deviation basis, is used, values of δ^2^H (‰) are relative to that for V-SMOW (Vienna Standard Mean Ocean Water).

#### GC-IRMS analysis of δ^13^C of chloromethane

In contrast to δ^2^H_CH__3__Cl_ analysis, chloromethane was combusted to carbon dioxide to measure the carbon isotope ratios of CH_3_Cl (δ^13^C_CH__3__Cl_). Therefore, a HP6890 GC (GasPro column) was interfaced with an Isoprime IRMS via a 850°C GCV combustion reactor containing a 0.65 mm i.d. quartz tube packed with small copper oxide rods (Cu(II)O) (Isoprime). The GC was held isothermal at 100°C and the helium flow entering the GC was 1.3 mL min^−1^.

A tank of ultrahigh-purity carbon dioxide with a known δ^13^C value of −36.2‰ was used as the working reference gas. Values of δ^13^C (‰) are relative to that for V-PDB (Vienna Pee Dee Belemnite).

All sample results were corrected for area and day-to-day drift in the system using a local CH_3_Cl reference (refer to Greule et al. (2012) for calibration) that was measured several times per day. The stable hydrogen and carbon isotope values for the local CH_3_Cl reference were −117.1‰ ± 4.7 ‰ and −30.34 ‰± 0.19 ‰, respectively.

Carbon and hydrogen isotope fractionations associated with chloromethane degradation by *M. extorquens* CM4 and *Hyphomicrobium* sp. MC1 were determined from the slopes (*b*_*C*_ *and b*_*H*_) of the linear regression of isotope variation (δ^13^C and δ^2^H) in chloromethane on the logarithm of the remaining chloromethane concentration (ln f):


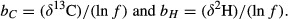


Fractionation factors α_C_ and α_H,_ corresponding to kinetic isotope effects were calculated as α = 1000/(*b* + 1000), and also reported as isotope enrichment factors (ε_C_ and ε_H_), calculated as ε = (α–1)10^3^. Error represents 95% confidence intervals calculated on the least-squares regression.

## Results

**Figure 1:**
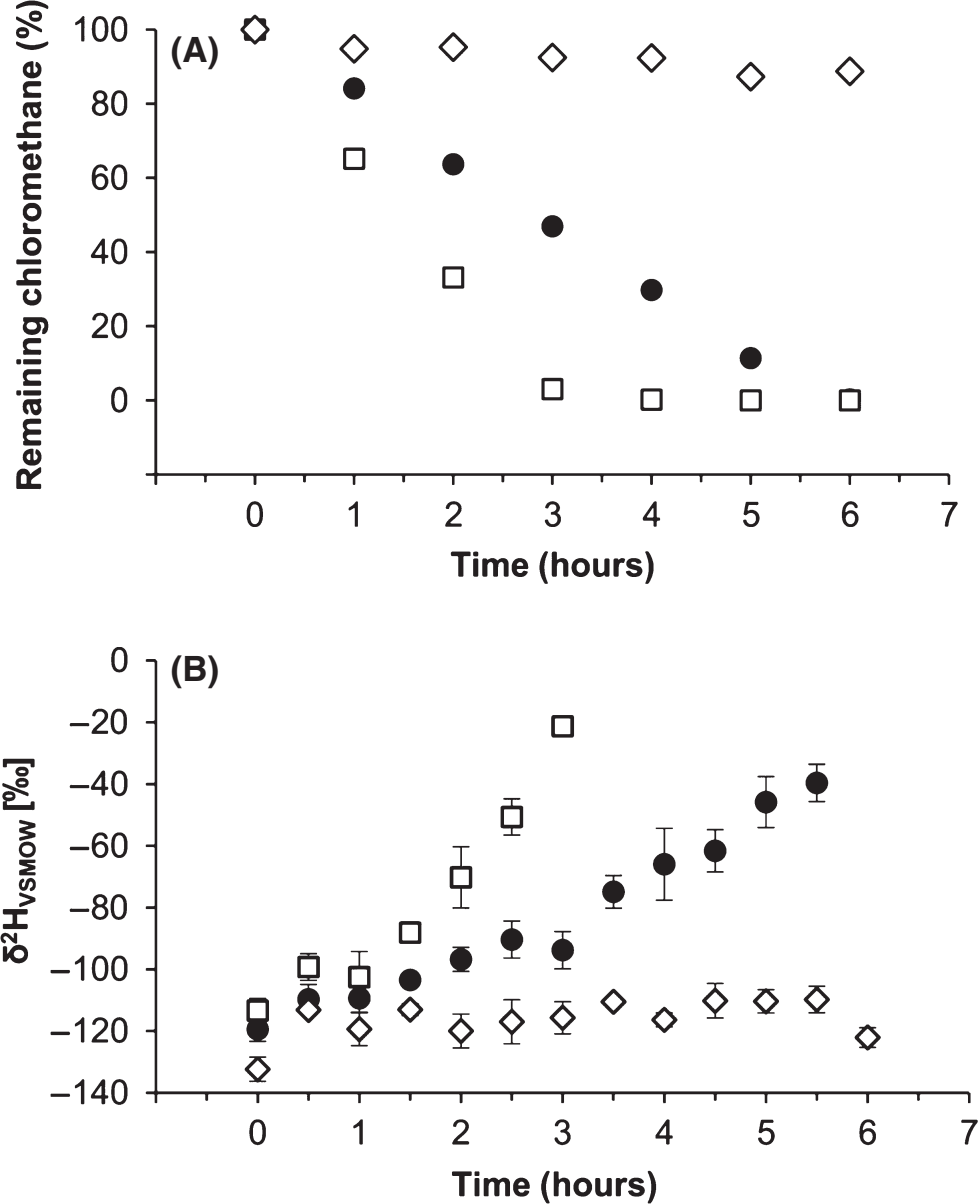
Hydrogen isotopic fractionation and degradation of chloromethane in bacterial cell suspensions. Consumption (A) and hydrogen isotope composition (B) of chloromethane. *Methylobacterium extorquens* CM4 (•), *Hyphomicrobium* sp. MC1 (⋄), abiotic control (□). Error bars indicate the standard deviation of three replicate measurements (*n* = 3).

Isotope enrichment experiments were performed with cell suspensions prepared from cultures of strains grown with chloromethane (10 mmol L^−1^) as the sole source of carbon and energy. All incubations were performed at 30°C with 10 mmol L^−1^ of chloromethane, and were stopped when chloromethane was fully consumed (after up to 6 h incubation). For commercial chloromethane gas (Fluka), values of δ^13^C and δ^2^H were determined as −31.31‰ ± 0.28‰ versus V-PDB and −124.2‰ ± 3.9‰ versus V-SMOW, respectively. Gas chromatographic analysis (Fig. 1A) showed that chloromethane was fully degraded within the duration of the incubation experiment by cell suspensions of chloromethane-degrading strains CM4 and MC1 under the investigated conditions. This was confirmed by measurements of chloride concentration in supernatants of cell suspensions at the end of the incubation period (9.00 ± 0.03 mmol L^−1^ and 9.25 ± 0.04 mmol L^−1^ for CM4 and MC1, respectively). The discrepancy with the expected value of 10 mmol L^−1^ basing on provided chloromethane is ascribed to losses in abiotic controls (11% and 8%, respectively), with no significant change in the observed δ^2^H value (Fig. 1). Independent incubation experiments were performed for determination of hydrogen and carbon isotope fractionation.

### Hydrogen isotope fractionation

A moderate enrichment of ^2^H in residual chloromethane upon incubation with cell suspensions of strains CM4 and MC1 was observed, with the δ^2^H value increasing from −124.2‰ ± 3.9‰ up to −39.6‰ ± 9.7‰ and −21.3‰ ± 15.3‰ for strains CM4 and MC1, respectively (Fig. 1B).

**Figure 2:**
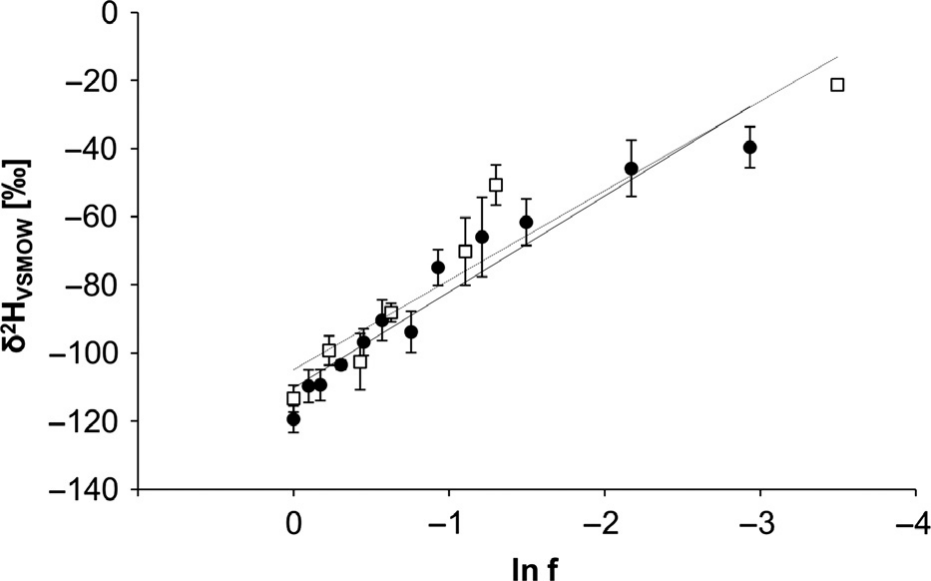
δ^2^H of chloromethane in relation to the fraction of remaining chloromethane (f) during incubation of bacterial cell suspensions with chloromethane. Lines represent best-fit linear regressions. *Methylobacterium extorquens* CM4 (•, solid line) and *Hyphomicrobium* sp. MC1 (□, dashed line). Error bars indicate the standard deviation of three replicate measurements (*n* = 3).

The hydrogen stable isotope enrichment factor (ε_H_) was calculated from the variation in chloromethane δ^2^H as a function of the natural logarithm of its remaining fraction (ln *f*) (Fig. 2), yielding closely similar values of kinetic isotopic effects α_H_ = 1.029 (with enrichment factor of ε_H_ = −29‰ ± 6‰) and α_H_ = 1.027 (ε_H_ = −27 ± 10‰) for hydrogen for strains CM4 and MC1, respectively (Table 1).

**Table 1:** Isotopic enrichment factor for carbon (ε_C_) and hydrogen (ε_H_) upon dehalogenation of chloromethane

	ε_C_ (‰)	*R*² 1	α_C_	ε_H_ (‰)	*R*²^1^	α_H_
*Methylobacterium extorquens* CM4	−41 ± 5^2^	0.9638	1.041	−29 ± 6	0.9298	1.029
*Hyphomicrobium* sp. MC1	−38 ± 3	0.9851	1.038	−27 ± 10	0.8985	1.027

1 Quality of fit to linear least-squares regression.

2 95% confidence interval calculated by linear least-squares regression.

### Carbon isotope fractionation

**Figure 3:**
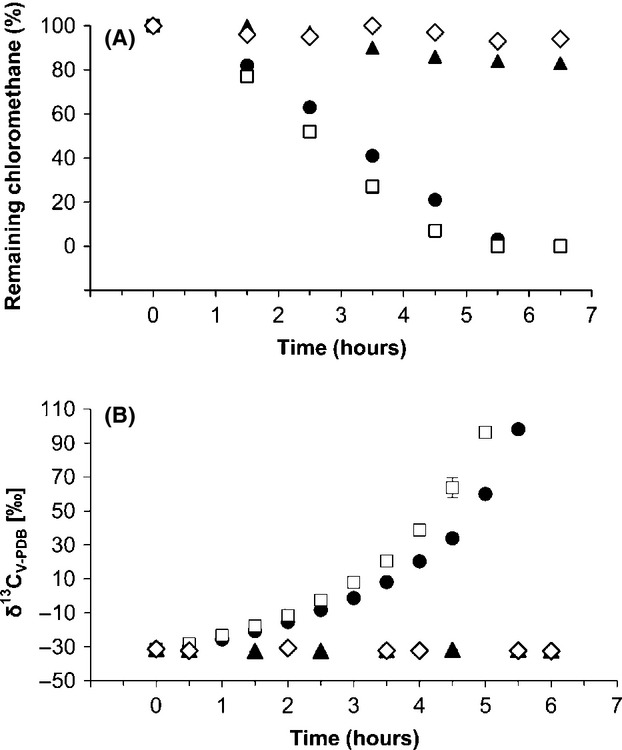
Carbon isotopic fractionation and degradation of chloromethane in bacterial cell suspensions. Consumption (A) and carbon isotope composition (B) of chloromethane. *Methylobacterium extorquens* CM4 (•), *Hyphomicrobium* sp. MC1 (□), abiotic control (⋄), and *Methylobacterium extorquens* AM1 (▲). Error bars indicate the standard deviation of three replicate measurements (*n* = 3).

**Figure 4:**
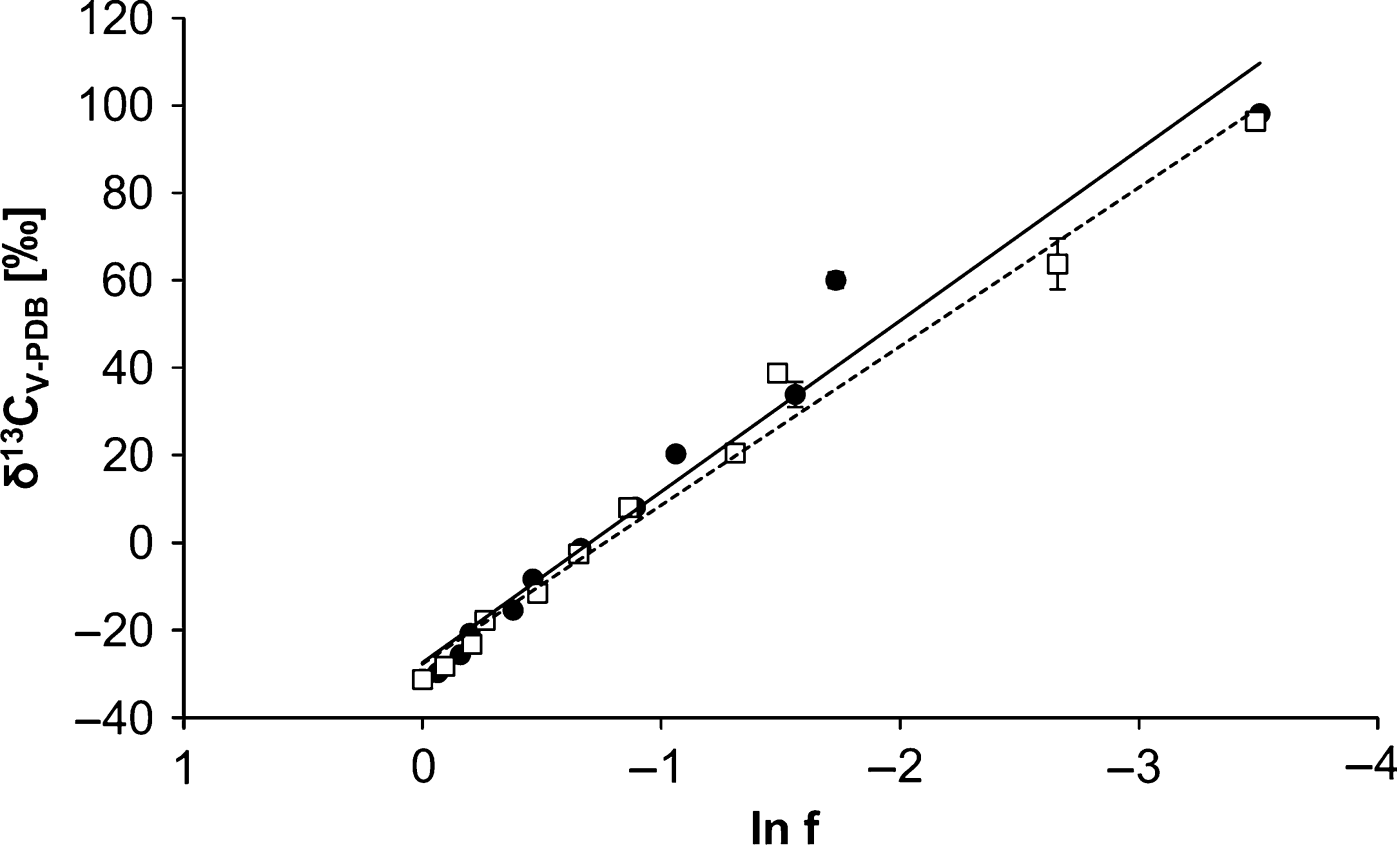
δ^13^C of chloromethane in relation to the fraction of remaining chloromethane (f) during incubation of bacterial cell suspensions. Lines represent best-fit linear regressions. *Methylobacterium extorquens* CM4 (•, solid line) and *Hyphomicrobium* sp. MC1 (□, dashed line). Error bars indicate the standard deviation of three replicate measurements (*n* = 3).

The observed δ^13^C of residual chloromethane increased from −31.31‰ up to 98.13‰ and 96.38‰ upon degradation of chloromethane by chloromethane-degrading strains CM4 and MC1, respectively (Fig. 3). In control experiments performed under identical conditions, little loss of chloromethane (6%) was observed both in a bacteria-free incubation, and was higher in a control incubation (17% chloromethane loss, Fig. 3A) with *Methylobacterium extorquens* AM1, a strain unable to degrade chloromethane but closely related to strain CM4 (Vuilleumier et al. 2009; Marx et al. 2012), possibly due to methylation of AM1 biomass. However, values of δ^13^C for chloromethane in both abiotic and biotic controls remained constant in these control experiments (Fig. 3B). Carbon kinetic isotope effects were calculated as described above for hydrogen (Fig. 4). Again, very similar values of kinetic isotope effects α_C_ = 1.041 (ε_C_ = −41‰ ± 5‰) and α_C_ = 1.038 _(_ε_C_ = −38‰ ± 3‰) were obtained for strains CM4 and MC1, respectively (Table 1).

## Discussion

Both chloromethane-degrading bacterial strains selected for this study, *Methylobacterium extorquens* CM4 and *Hyphomicrobium* sp. strain MC1, as well as the two previously investigated *Aminobacter* strains (Miller et al. 2001), grow with chloromethane using the well-characterized methyltransferase-dependent *cmu* pathway (Studer et al. 2002; Nadalig et al. 2011). We showed here that degradation of chloromethane by strains CM4 and MC1 is accompanied by quite similar isotopic fractionation of carbon and hydrogen elements (Table 1). Moreover, a two-dimensional presentation of the δ^13^C versus δ^2^H values obtained at the same extent of chloromethane degradation yields the same slope for both strains (Fig. S2), as expected, if the same transformation mechanism is involved (Elsner et al. 2007).

For carbon, results were closely similar to those previously observed for *A. ciceronei* IMB-1 (ε = −47‰) and *A. lissarensis* CC495 (ε = −42‰) (Miller et al. 2001). Larger differences in stable carbon isotope enrichment factors were observed for degradation of another chlorinated methane, dichloromethane, by different strains using the same enzyme (Nikolausz et al. 2006). For example, values of ε = −49‰ for *Hyphomicrobium* sp. GJ21 and ε = −62‰ for *Methylobacterium extorquens* DM4 were obtained in whole-cell experiments despite very similar kinetic parameters and amino acid sequences of the corresponding glutathione S-transferase/dichloromethane dehalogenase enzyme variants (Vuilleumier et al. 2001). By comparison, chloromethane dehalogenase proteins show a much larger sequence diversity (Nadalig et al. 2011). Although different enzymatic systems are involved, dehalogenation of both chloromethane and dichloromethane features a similar S_N_2-type cofactor-catalyzed reaction (cobalamin for chloromethane dehalogenase and glutathione for dichloromethane dehalogenase). It was suggested that the observed large difference in dichloromethane isotope enrichment between strains GJ21 and DM4 was due to amino acid differences close to the substrate-binding site of DCM dehalogenase (Nikolausz et al. 2006). No X-ray structures for chloromethane and dichloromethane dehalogenases are yet available, so the molecular basis of the observed differences in carbon isotope fractionation for chloromethane dehalogenase and dichloromethane dehalogenase can only be speculated upon at this point.

As for carbon isotope enrichment factors, hydrogen isotope enrichment factors for chloromethane upon bacterial degradation, evaluated for the first time in this study, were similar for the two strains investigated. Clearly, hydrogen isotope fractionation for chloromethane was less pronounced (−27‰ to −29‰) than for carbon. Isotope fractionation is usually larger for hydrogen than for carbon because of the larger relative mass difference between heavy and light isotopes of hydrogen. For example, isotope effects were three to eight times more important for hydrogen than for carbon in the case of bacterial degradation of toluene (Hunkeler et al. 2001), benzene (Morasch et al. 2001), and methane (Feisthauer et al. 2011). In this case, however, no C–H bond is broken upon dehalogenation, the first step of chloromethane degradation. Consequently, only carbon and chlorine atoms of the C–Cl bond are subject to primary isotope effects, and the rather low fractionation of hydrogen compared to carbon is most likely due to secondary isotope effects only.

Stable carbon isotope analysis has already proved to be a powerful tool in investigations of the atmospheric budget of chloromethane (Thompson et al. 2002; Gola et al. 2005; Keppler et al. 2005; Saito and Yokouchi 2008; Greule et al. 2012). Hydrogen isotope fractionation measurements, when applied to methyl halides at ambient atmospheric concentrations, hold the promise to complete our knowledge of the chloromethane cycle, most particularly with respect to its biological aspects. The data reported here are relevant to the bacterial sink for chloromethane, and will contribute to constrain the global chloromethane budget alongside recent estimates of strongly negative stable hydrogen isotope ratios for chloromethane sources from dry halophytic plants, living vegetation, and biomass burning (Greule et al. 2012), which represent the bulk fraction (>90%) of chloromethane in the atmosphere. Sinks of chloromethane in the environment are dominated by reaction of chloromethane with hydroxyl radicals in the atmosphere and by biological degradation by microorganisms in soils, but the relative contributions of these two processes are highly uncertain (Keppler et al. 2005). The carbon and hydrogen isotope enrichment factors found for reaction of chloromethane with OH radicals were reported to be −59‰ ± 8‰ and −410‰ ± 50‰, respectively (Gola et al. 2005; Sellevåg et al. 2006). The carbon isotope enrichment factor for chloromethane-degrading bacteria was also found to be large, ranging from −38‰ to −47‰ (Miller et al. 2001, this work). In contrast, however, hydrogen isotope enrichment factors of chloromethane degradation by microorganisms are modest (this study) and thus might have a minor effect on atmospheric δ^2^H values. Thus, a 2D isotope approach including both isotope signatures (δ^2^H and δ^13^C) of sources and sinks of chloromethane will certainly assist to better constrain the global budget in the near future, and in particular provide a deeper insight into the importance (global sink strength) of the microbial sink of chloromethane. Values of isotopic fractionation of both hydrogen and carbon of chloromethane for chloromethane-degrading bacteria determined in this work thus represents a further step on the way to refining models of the global chloromethane cycle.

## Supporting Information

**Figure S1.** Chloromethane dehalogenation in strains *Methylobacterium extorquens* CM4 and *Hyphomicrobium* sp. MC1 requires a two-domain corrinoid-dependent methyltransferase CmuA and a homodimeric tetrahydrofolate-dependent methyltransferase CmuB, as demonstrated previously for strain CM4 (see Studer et al. (2001) for details).

**Figure S2.** Changes in carbon and hydrogen isotope ratios for degradation of chloromethane by *M. extorquens* CM4 (•, solid line) and *Hyphomicrobium* sp. MC1 (□, dashed line).
