# Supplementary figures and images for: Hydrogen and carbon isotope fractionation during degradation of chloromethane by methylotrophic bacteria
Source: Microbiologyopen. 2013 Sep 8;2(6):893–900. doi: 10.1002/mbo3.124 (PMC3892336; doi:10.1002/mbo3.124)

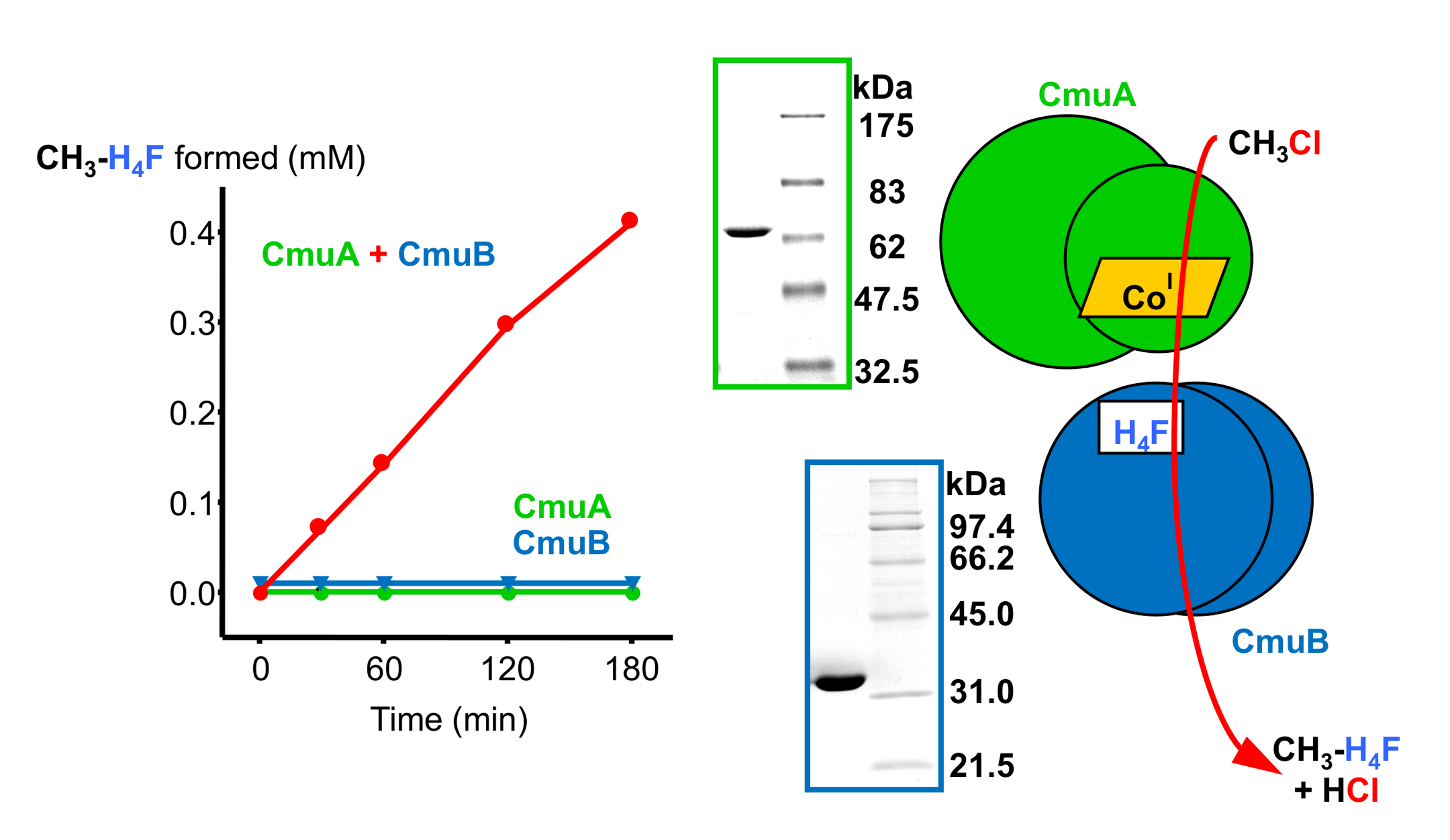

Supplement: Supplementary file 1 [file mbo30002-0893-SD1.tif]

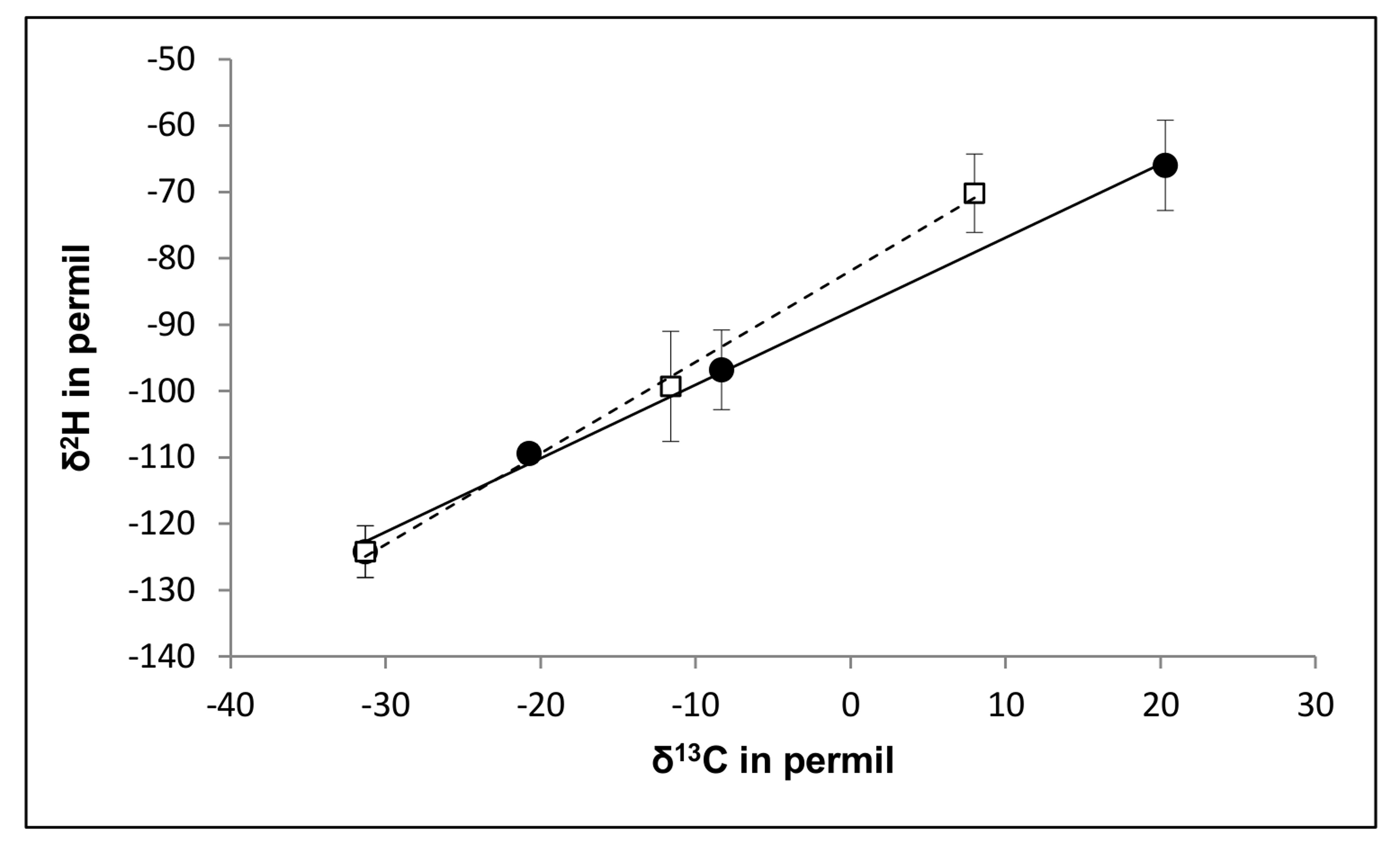

Supplement: Supplementary file 2 [file mbo30002-0893-SD2.tif]
